# Synaptic defects in a drosophila model of muscular dystrophy

**DOI:** 10.3389/fncel.2024.1381112

**Published:** 2024-05-15

**Authors:** Jessica M. Sidisky, Alex Winters, Russell Caratenuto, Daniel T. Babcock

**Affiliations:** ^1^Department of Biological Sciences, Lehigh University, Bethlehem, PA, United States; ^2^Department of Brain and Cognitive Sciences, The Picower Institute for Learning and Memory, Cambridge, MA, United States; ^3^Department of Biology, Massachusetts Institute of Technology, Cambridge, MA, United States

**Keywords:** neuromuscular junction, dystrophin, flight, presynaptic, postsynaptic, degeneration

## Abstract

Muscular dystrophies are a devastating class of diseases that result in a progressive loss of muscle integrity. Duchenne Muscular Dystrophy, the most prevalent form of Muscular Dystrophy, is due to the loss of functional Dystrophin. While much is known regarding destruction of muscle tissue in these diseases, much less is known regarding the synaptic defects that also occur in these diseases. Synaptic defects are also among the earliest hallmarks of neurodegenerative diseases, including the neuromuscular disease Amyotrophic Lateral Sclerosis (ALS). Our current study investigates synaptic defects within adult muscle tissues as well as presynaptic motor neurons in Drosophila *dystrophin* mutants. Here we demonstrate that the progressive, age-dependent loss of flight ability in *dystrophin* mutants is accompanied by disorganization of Neuromuscular Junctions (NMJs), including impaired localization of both presynaptic and postsynaptic markers. We show that these synaptic defects, including presynaptic defects within motor neurons, are due to the loss of Dystrophin specifically within muscles. These results should help to better understand the early synaptic defects preceding cell loss in neuromuscular disorders.

## 1 Introduction

Muscular Dystrophy (MD) is comprised of a large group of more than 30 inherited diseases that are primarily characterized by the progressive loss of skeletal muscle integrity (Emery, 2002). Duchenne Muscular Dystrophy (DMD), which is the most common form of Muscular Dystrophy, is caused by mutations in the *dystrophin* gene (Hoffman et al., 1987). Dystrophin is a component of the Dysotrophin glycoprotein complex, which serves as an integral link between the cytoskeleton and the plasma membrane of muscle fibers (Ervasti and Campbell, 1991). Disruption of this link results in the loss of the structural integrity of muscles and eventual cell death. There is no current cure for Muscular Dystrophy, further highlighting the need to better understand the cellular and molecular mechanisms underlying this group of diseases.

While the progressive loss of muscle integrity has been the major area of focus for muscular dystrophies, studies have demonstrated early synaptic defects at NMJs that precede neuronal loss in these disorders. Disorganization of the postsynaptic membrane was found in Dystrophin-deficient mice (Shiao et al., 2004), along with a decrease in the density of postsynaptic acetylcholine receptors (Rafael et al., 2000).

Studies in *Drosophila* have also been useful for understanding the cellular and molecular mechanisms underlying Muscular Dystrophies along with other neuromuscular disorders (Kreipke et al., 2017). Dystrophin, for example, is well conserved between Drosophila and mammals (Neuman et al., 2001), allowing the vast genetic toolkit available in flies to be used to help better understand the mechanisms underlying DMD. Indeed, transgenic knockdown of Dystrophin isoforms in muscles revealed roles for *Drosophila* Dystrophin in maintaining muscle cell integrity (van der Plas et al., 2007) as well as regulating neurotransmitter release from presynaptic motor neurons (van der Plas et al., 2006). Additionally, genetic modifier screens have identified novel interactions involving dystrophin as well as other components of the Dystrophin Glycoprotein Complex (Kucherenko et al., 2011).

Here we investigate the progressive loss of synaptic integrity in adult Drosophila NMJs located within the Dorsal Longitudinal Muscles (DLMs) of the thorax as well as the Ventral Abdominal Muscles (VAMs) with age. Using *Dystrophin* mutants, we observe a progressive loss of flight ability that is accompanied by loss of synaptic integrity and disruption of pre- and post-synaptic markers within the indirect flight muscles. Within the abdominal body wall muscles, synaptic markers are similarly disrupted despite a lack of defects to gross synaptic morphology. Finally, we demonstrate that the synaptic defects observed in *Dystrophin* mutants are recapitulated by RNAi-mediated knockdown within muscle tissue, highlighting the requirement of Dystrophin within muscles to maintain presynaptic integrity. Altogether, these results help to provide a greater understanding of the earliest hallmarks of neuromuscular disorders.

## 2 Results

### 2.1 Locomotor and synaptic defects in *dystrophin* mutants

**FIGURE 1:**
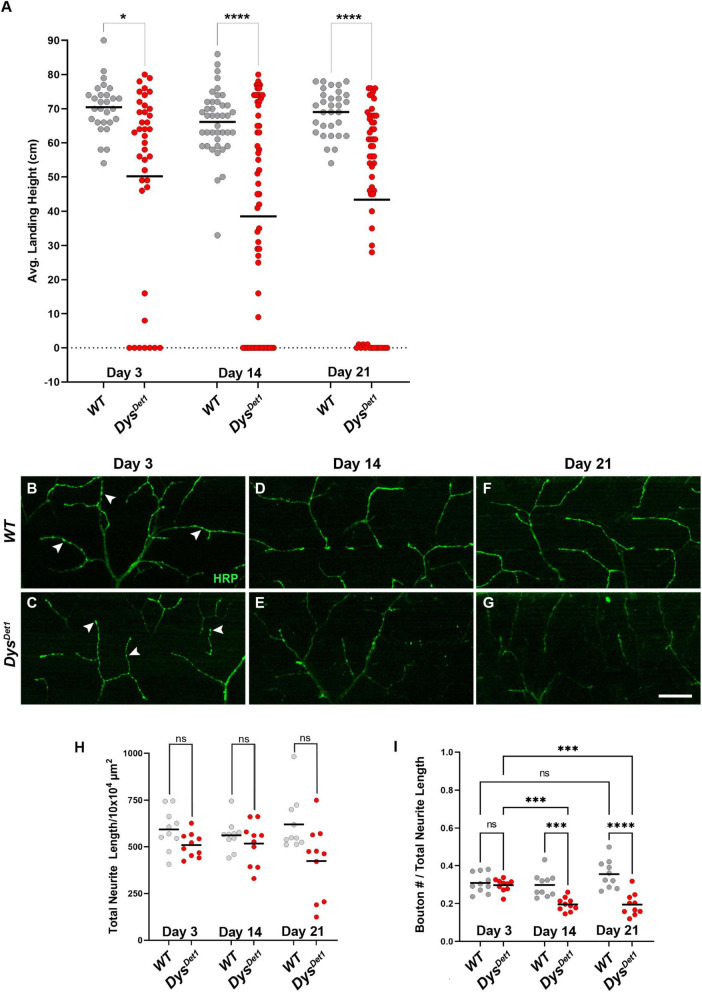
Flight defects accompany presynaptic defects in *dystrophin* mutants. **(A)** Flight ability of *dys*^*Det*1^ mutant flies (red dots) compared to *WT* controls (gray dots) as assessed by average landing height at Day 3, Day 14, and Day 21. Black lines represent the average landing height for each group. **(B–G)** DLM synaptic morphology of *WT* controls **(B,D,F)** and *dys*^*Det*1^ mutants **(C,E,G)** using HRP staining (green). Arrowheads highlight individual synaptic boutons. **(H)** Total neurite length at NMJs for *WT* and *dys*^*Det*1^ mutants. **(I)** Bouton density as measured along neurite length for *WT* and *dys*^*Det*1^ mutants. Scale Bar in panel **(G)** is 20 μm for panels **(B–G)**. ****p < 0.0001; ***p < 0.001; *p < 0.05; n.s., not significant, using one-way ANOVA with Tukey’s *Post hoc* test for multiple comparisons. Flight ability was assessed for each group in triplicate.

To assess the functional and structural integrity of NMJs in *dystrophin* mutants, we compared *dys*^*Det*–1^ mutants and WT controls. *dys*^*Det*–1^ is a spontaneous mutation that was isolated in 1935. It was originally characterized for the “detached” phenotype defined by the disruption of crossveins in the wing, and was later mapped to a mutation in Dystrophin and characterized as a hypomorphic allele (Christoforou et al., 2008). We first measured flight ability of *dys*^*Det*–1^ mutants and WT controls at Day 3, 14, and 21. While WT flies maintained a robust flight performance at all ages tested, the flight ability of *dys*^*Det*–1^ mutants progressively worsened with age (Figure 1A). The defect in flight ability of *dys*^*Det*–1^ mutants was significant even at the earliest measured time point of Day 3, and this locomotor impairment became more severe by Day 14. In contrast, WT flight ability remained consistent even at Day 21.

We next examined whether the flight defect in *dys*^*Det*–1^ mutants was accompanied by changes to NMJ morphology. Using Horseradish Peroxidase (HRP) to label neuronal membranes, we measured total neurite length within the Dorsal Longitudinal Muscles (Figures 1B–G). We found no significant difference in neurite length between *dys*^*Det*–1^ mutants and WT controls at Day 3, 14, or 21 (Figure 1H), suggesting that the defective flight ability in *dys*^*Det*–1^ mutants is not due to a change in gross morphology of motor neuron terminals. Upon closer inspection, however, we noticed that the axon terminals of older *dys*^*Det*–1^ mutants appeared to have fewer “en passant” boutons that are characteristic of these NMJs (Hebbar and Fernandes, 2004). While measuring bouton density, we found no significant differences in bouton density between *dys*^*Det*–1^ mutants and WT controls at Day 3. However, bouton density became progressively sparser in *dys*^*Det*–1^ mutants compared to controls at Day 14 and Day 21 (Figure 1I). Together, these results demonstrate disruption of presynaptic architecture in the form of bouton loss that accompanies the progressive loss of flight ability in *dystrophin* mutant flies.

### 2.2 Progressive loss of active zones in *dystrophin* mutant motor neurons

**FIGURE 2:**
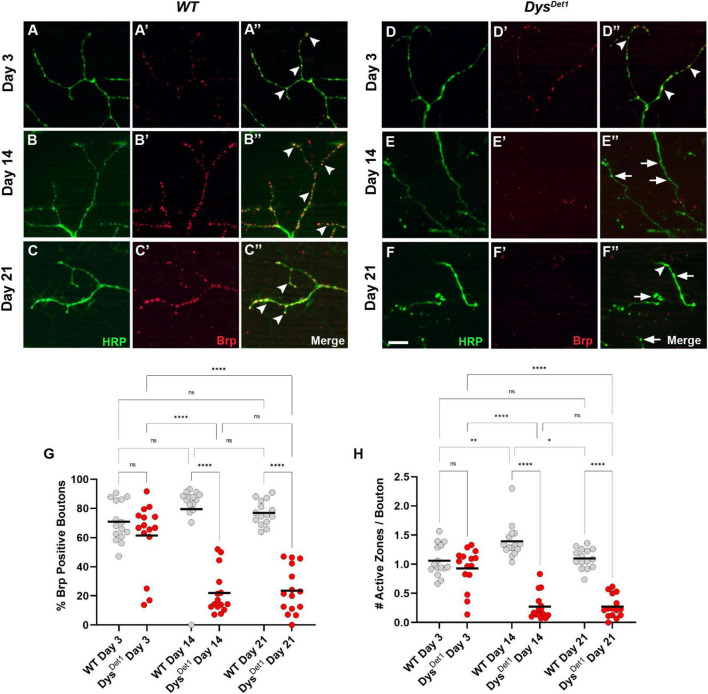
*dystrophin* mutants display a progressive loss of active zones. **(A–F)** Active zone distribution in WT **(A–C)** and *dys*^*Det*1^ mutant flies **(D–F)** at Day 3, Day 14, and Day 21. Active zones are marked using anti-bruchpilot (Brp) (red). Arrowheads mark synaptic boutons (HRP, green) that contain at least one active zone. Arrows highlight boutons that lack Brp staining. **(G)** Quantification of boutons that contain at least one active zone in *dys*^*Det*1^ mutants (red dots) compared to *WT* controls (gray dots) at Day 3, Day 14, and Day 21. Black lines represent the average value for each group. **(H)** Quantification of the number of active zones located within individual synaptic boutons for each group. Scale Bar in panel **(F)** is 10 μm for panels **(A–F)**. ****p < 0.0001; ***p < 0.001; **p < 0.01; *p < 0.05; n.s., not significant, using one-way ANOVA with Tukey’s *Post hoc* test for multiple comparisons.

To further examine the structural integrity of neuromuscular synapses in *dystrophin* mutants, we next examined the distribution of active zones within synaptic boutons. Active zones represent the specific regions where neurotransmitters are released from presynaptic neurons, and these structures are often labeled in *Drosophila* using the active zone marker Bruchpilot (Brp) (Kittel et al., 2006; Wagh et al., 2006). Bouton size within the Dorsal Longitudinal Muscles (DLM) is relatively small, with most boutons containing 1 or 2 active zones (Kawasaki et al., 2004). To assess active zone distribution in *dystrophin* mutants, we first measured the frequency of Brp-positive boutons in *dys*^*Det*–1^ mutants compared to WT controls (Figures 2A–G). At early adult stages, the majority of synaptic boutons contain at least one active zone (Figures 2A, D, arrowheads), with no significant difference between *dys*^*Det*–1^ and WT synapses. However, most synaptic boutons lack Brp staining by Day 14 in *dys*^*Det*–1^ mutants (Figures 2B, E, arrows), with a similar deficit seen at Day 21 (Figures 2C, F). This suggests that active zones are disrupted even among the remaining boutons in *dys*^*Det*–1^ mutants.

We also specifically compared the number of active zones per bouton in *dys*^*Det*–1^ mutants and WT controls. At Day 3, the average number of active zones per bouton did not significantly differ (Figure 2H). In WT flies, this value slightly increases by Day 14. In contrast, the number of active zones per bouton significantly decreases by this time point and remains consistent at Day 21. Together, these results highlight the progressive disruption of active zones as part of the presynaptic dysfunction in *dys*^*Det*–1^ mutant flies.

### 2.3 Impairment of abdominal NMJs in dystrophin mutants

While neuromuscular synapses within the indirect flight muscles are impaired in *dystrophin* mutants, we also examined whether other adult NMJs are similarly affected. Specifically, we examined NMJs located within the Ventral Abdominal Muscles (VAMs). These NMJs have been observed in the context of aging, and the majority of presynaptic and postsynaptic markers used for larval NMJ analysis work well here (Hebbar et al., 2006; Wagner et al., 2015; Pons et al., 2017; Wagner, 2017; Banerjee et al., 2021).

**FIGURE 3:**
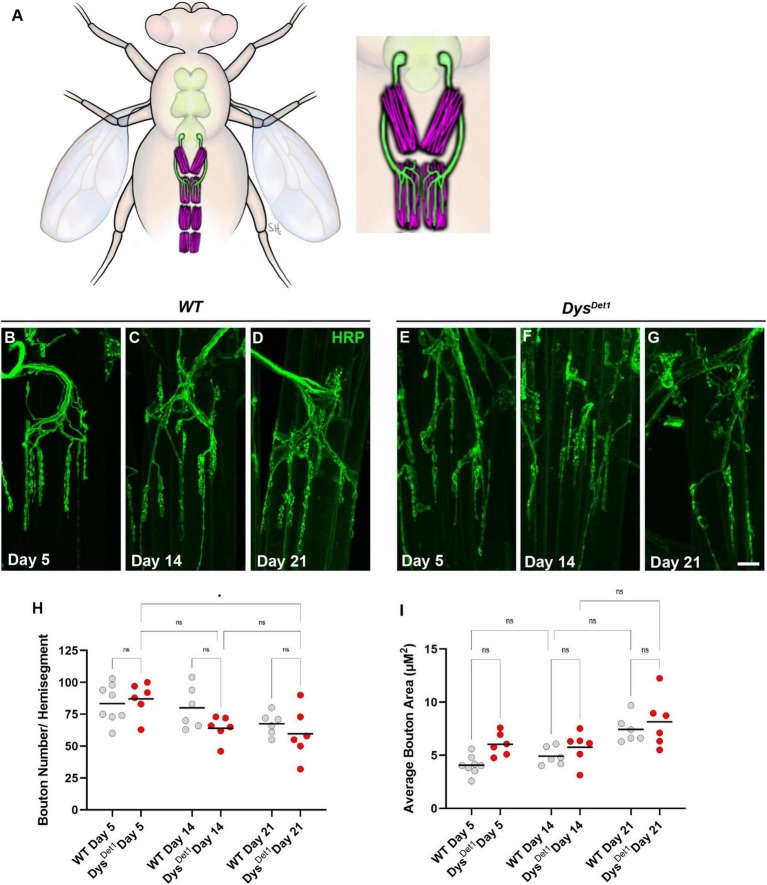
Morphology of abdominal NMJs in *dystrophin* mutants. **(A)** Illustration of Ventral Abdominal Muscles and motor neuron innervation. Each abdominal segment contains a symmetrical pair of muscles, with each muscle receiving innervation from a single motor neuron. Innervation is only shown for segment A3 for simplicity. Inset highlights the innervation pattern of muscles in segment A3, which is used in this study. **(B–G)** NMJ morphology of Ventral Abdominal Muscles in WT **(B–D)** and *dys*^*Det*1^ mutants **(E–G)**. **(H)** Quantification of the average number of boutons within each hemi-segment in *dys*^*Det*–1^ mutants (red dots) compared to *WT* controls (gray dots) at Day 5, Day 14, and Day 21. **(I)** Average bouton size for *dys*^*Det*1^mutants and WT controls at Day 5, Day 14, and Day 21. Scale Bar in panel **(G)** is 10 μm for panels **(B–G)**. *p < 0.05; n.s., not significant, using one-way ANOVA with Tukey’s *Post hoc* test for multiple comparisons.

The VAMs are located along the ventral midline of the abdomen, with one pair of muscles within each abdominal segment (Pons et al., 2017; Figure 3A). Each muscle within a segment is innervated by a single motor neuron that originates in the thoracic ganglion (Figure 3A). In contrast to the synapses located within the indirect flight muscles, the NMJs within the ventral abdominal muscles more closely resemble the well-studied NMJs innervating the larval body wall muscles. Shared features include relatively larger bouton size compared to the DLMs, each with several active zones, and a well-defined subsynaptic reticulum that surrounds each bouton (Hebbar et al., 2006; Wagner et al., 2015; Wagner, 2017).

We first assessed the gross morphology of abdominal NMJs by measuring the number and size of synaptic boutons in both WT and *dys*^*Det*–1^ mutants at Day 5, Day 14, and Day 21 (Figures 3B–G). Because some adult motor neuron terminals are not stabilized until a few days after eclosion (Rivlin et al., 2004), we used Day 5 as the earliest timepoint for measurements. When comparing the number of boutons present within each muscle, we did not observe many obvious differences between WT and *dys*^*Det*–1^ mutants (Figure 3H). We did measure a small, yet significant, reduction in bouton number in *dys*^*Det*–1^ mutants between Day 5 and Day 21. However, there were no significant differences between WT and *dys*^*Det*–1^ mutants at any given timepoint. For bouton size, we noticed a trend of increasing average bouton area with age. As with bouton number, we also noticed a similar trend of larger bouton size in both WT and *dys*^*Det*–1^ mutants with no significant differences between them. Together, these results suggest that the gross morphology of *dys*^*Det*–1^ mutant abdominal NMJs is not significantly impaired, in contrast to the morphology defects observed within the indirect flight muscles.

**FIGURE 4:**
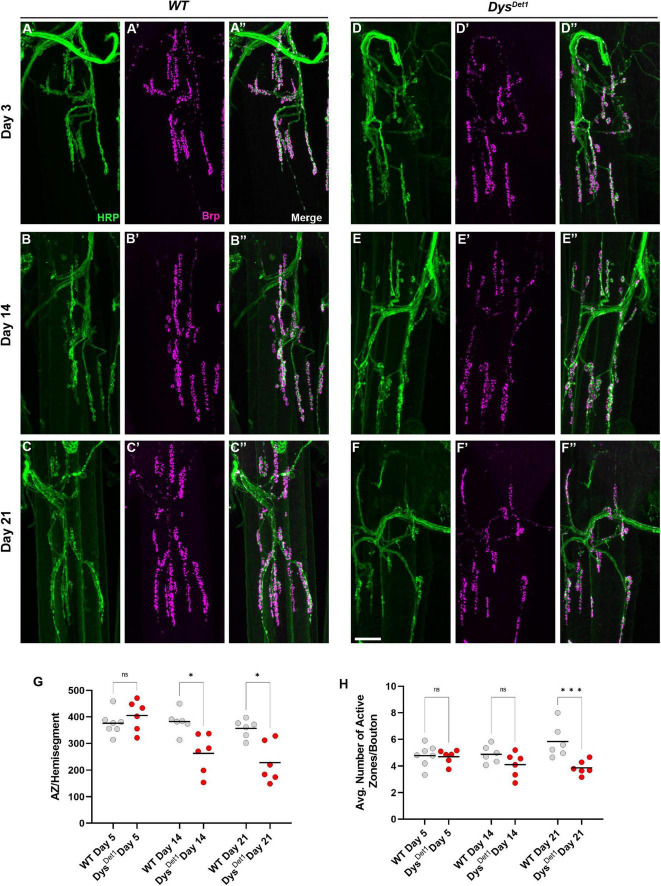
Disruption of active zone distribution at abdominal NMJs in *dystrophin* mutants. **(A–F)** Active zone distribution at abdominal NMJs in WT **(A–C)** and *dys*^*Det*–1^ mutants **(D–F)**. Individual active zones are marked by Brp + puncta (magenta). **(G)** Average number of active zones located within each individual muscle (hemi-segment) in *dys*^*Det*1^ mutants (red dots) compared to *WT* controls (gray dots) at Day 5, Day 14, and Day 21. **(H)** Average number of active zones within each synaptic bouton at Day 5, Day 14, and Day 21. Scale Bar in panel **(F)** is 10 μm for panels **(A–F)**. ***p < 0.001; *p < 0.05; n.s., not significant, using one-way ANOVA with Tukey’s *Post hoc* test for multiple comparisons.

We next assessed the distribution of active zones within synaptic boutons at abdominal NMJs. Active zones were labeled using anti-Bruchpilot (Brp) staining. As with the measurements for gross morphology, both WT and *dys*^*Det*–1^ mutants were measured at Day 5, Day 14, and Day 21 (Figures 4A–F). At Day 5, *dys*^*Det*–1^ mutants displayed a similar number of active zones per muscle (or hemi-segment) compared to WT flies (Figures 4A, D, G). However, the number of active zones decreased in *dys*^*Det*–1^ mutants by Day 14, and this progressed further by Day 21 (Figures 4B–G). We also measured the average number of active zones within each synaptic bouton under the same conditions. While the number of active zones per bouton is initially similar between WT and *dys*^*Det*–1^ mutants at early time points, *dys*^*Det*–1^ mutants have significantly fewer active zones within each bouton by Day 21. These results highlight a presynaptic defect in *dys* mutants despite relatively normal synaptic morphology. This could suggest that maintaining active zone integrity is more sensitive to mutations in *dys* than gross synaptic morphology.

**FIGURE 5:**
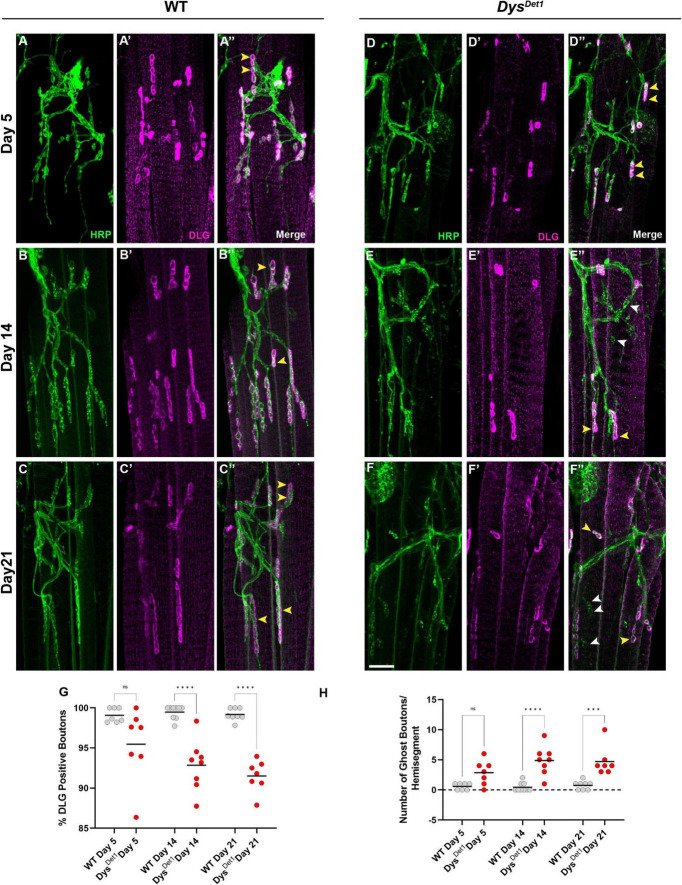
Impaired postsynaptic integrity at abdominal NMJs in *dystrophin* mutants. **(A–F)** DLG labels the subsynaptic reticulum at abdominal NMJs in WT **(A–C)** and *dys*^*Det*1^ mutants **(D–F)**. **(G)** Percentage of HRP + boutons that are also DLG + (yellow arrowheads) in *dys*^*Det*1^ mutants (red dots) compared to *WT* controls (gray dots) at Day 5, Day 14, and Day 21. **(H)** Prevalence of ghost boutons, which are defined as HRP + but DLG- boutons (white arrowheads) in *dys*^*Det*1^ mutants (red dots) and *WT* controls (gray dots). Scale Bar in panel **(F)** is 10 μm for panels **(A–F)**. ****p < 0.0001; ***p < 0.001; n.s., not significant, using one-way ANOVA with Tukey’s *Post hoc* test for multiple comparisons.

We also measured the integrity of the postsynaptic membrane in abdominal NMJs. In contrast to the indirect flight muscles, the abdominal NMJs contain a distinct subsynaptic reticulum (SSR) (Wagner et al., 2015; Wagner, 2017), at which the muscle cell membrane surrounds synaptic boutons. We used the postsynaptic marker Disks Large (DLG) (Parnas et al., 2001) to assess the postsynaptic integrity at abdominal NMJs. We first measured the percentage of synaptic boutons that stained positive for DLG in WT and *dys*^*Det*–1^ mutants at Day 5, Day 14, and Day 21. At Day 5, we did not detect a significant difference in DLG-positive boutons between WT and *dys*^*Det*–1^ mutants (Figures 5A, D, G). However, by Day 14 there is a significant decrease in the percentage of DLG-positive boutons in *dys*^*Det*–1^ mutants. This phenotype progressively worsens by Day 21 (Figures 5B–G).

We also measured the number of “ghost boutons” present in WT and *dyt*^*Det*–1^ abdominal NMJs. Ghost boutons are characterized by the presence of presynaptic HRP staining but are not surrounded by the postsynaptic SSR (Guangming et al., 2020). Ghost boutons were rarely seen in our WT samples, and there was no significant difference in ghost bouton prevalence between WT and *dys*^*Det*–1^ mutants at Day 5 (Figure 5H). However, by Day 14 there was a significant increase in the number of ghost boutons in *dys*^*Det*–1^ mutants. This increase in ghost boutons was also seen at Day 21 (Figure 5H). During development of larval NMJs, the presence of ghost boutons is typically associated with the formation of immature boutons (Ataman et al., 2006; Piccioli and Littleton, 2014). However, the age-dependent presence of ghost boutons in adult NMJs could also indicate the progressive deterioration of postsynaptic structures. Altogether, these results highlight the progressive disruption of postsynaptic integrity in abdominal NMJs in *dystrophin* mutants.

### 2.4 Dystrophin expression in muscles is required to maintain presynaptic and postsynaptic integrity

Our results suggest that mutations in *Dystrophin* impair synaptic integrity within presynaptic motor neurons as well as postsynaptic muscle fibers. While most investigations into muscular dystrophy have focused on the role of Dystrophin within muscles, it is unclear whether a muscle-specific role of Dystrophin underlies the pre- and post-synaptic defects seen here. Recent studies have uncovered roles for both Dystrophin and Dystroglycan in the nervous system (Marrone et al., 2011; Nickolls and Bonnemann, 2018). We also recently completed a genome-wide screen for genes associated with synaptic maintenance that identified a variety of genes required in different tissues to maintain synaptic integrity (Sidisky et al., 2023). Several of these genes regulate NMJ maintenance in multiple cell types of the tripartite synapse, including presynaptic motor neurons, postsynaptic muscles, and associated glial cells.

**FIGURE 6:**
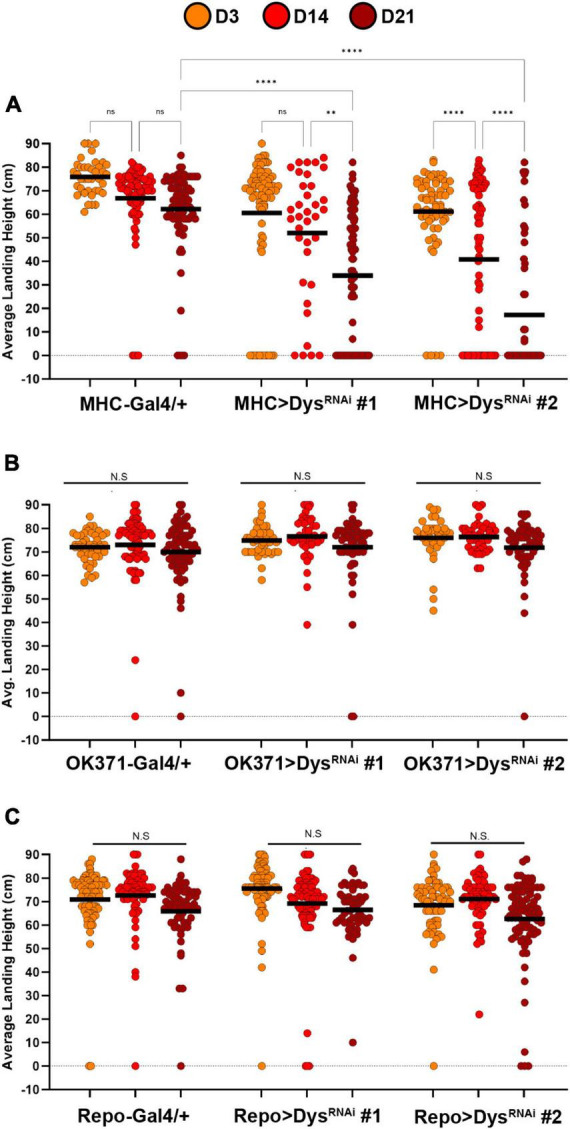
Knockdown of Dystrophin in muscle cells impairs flight ability. Flight ability of transgenic flies with tissue specific knockdown of Dystrophin in muscles using MHC-Gal4 **(A)**, Motor neurons using OK371-Gal4 **(B)**, and Glia using Repo-Gal4 **(C)**. Two independent RNAi transgenes (RNAi #1 and RNAi #2) were used for each condition. Circles indicate the landing height of individual flies at Day 3, Day 14, and Day 21. Black lines represent the average landing height for each group. ****p < 0.0001; **p < 0.01; n.s., not significant, using one-way ANOVA with Tukey’s *Post hoc* test for multiple comparisons. Flight ability was assessed for each group in triplicate.

To investigate the tissue-specific roles of Dystrophin on synaptic maintenance, we first knocked down Dystrophin using two independent RNAi transgenes in each component of the tripartite synapse and measured flight performance. Knockdown of Dystrophin in muscles using *MHC-Gal4* (Klein et al., 2014) resulted in a progressive loss of flight performance by Day 14 that continued at Day 21 (Figure 6A). The flight defects observed upon muscle-specific knockdown of Dystrophin are similar to those seen in *dys*^*Det*–1^ mutants (Figure 1), highlighting the requirement of Dystrophin in postsynaptic cells to maintain synaptic integrity. To test whether Dystrophin is also required in other synaptic cell types, we also knocked down Dystrophin in presynaptic motor neurons using *OK371-Gal4* (Meyer and Aberle, 2006). However, we did not observe any flight defects upon motor neuron-specific knockdown of Dystrophin (Figure 6B). Finally, we knocked down Dystrophin specifically in glial cells using *Repo-Gal4* (Sepp et al., 2001). Glial-specific knockdown of Dystrophin did not result in flight defects compared to controls (Figure 6C). Together, these results suggest that maintaining the functional integrity of neuromuscular synapses within the Indirect Flight Muscles requires postsynaptic expression of Dystrophin.

**FIGURE 7:**
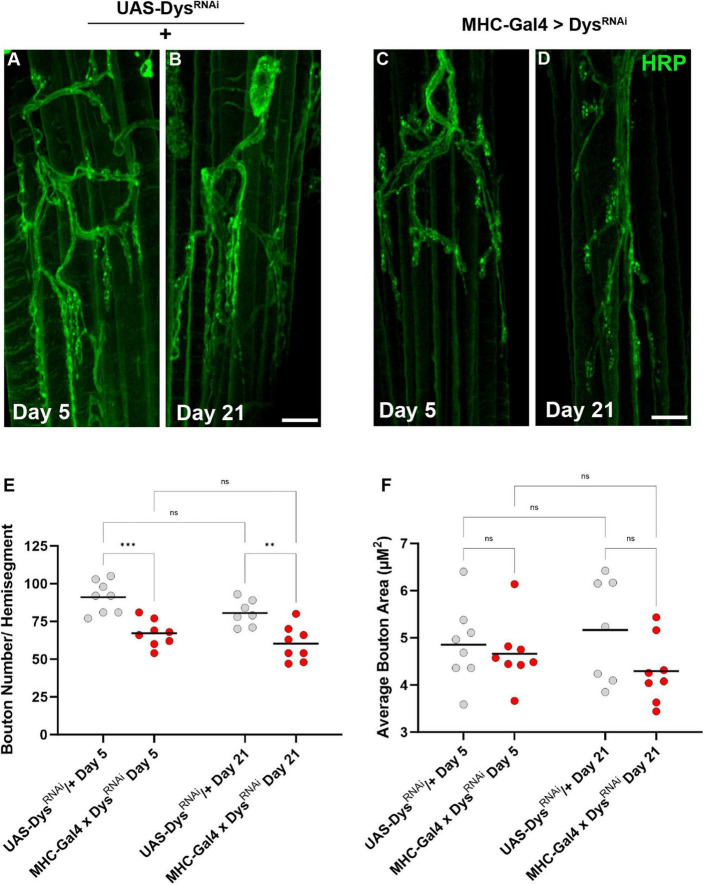
Abdominal NMJ gross morphology upon Dystrophin knockdown. **(A–D)** Abdominal NMJ morphology in control flies **(A,B)** and flies with muscle-specific knockdown of Dystrophin **(C,D)**. **(E)** Average number of boutons within each muscle cell with muscle-specific knockdown of Dystrophin (red dots) compared to *UAS/* + controls (gray dots) at Day 5 and Day 21. **(F)** Average bouton size upon Dystrophin knockdown and WT controls at Day 5 and Day 21. Scale Bar in panel **(D)** is 10 μm for panels **(A–D)**. ***p < 0.001; **p < 0.01; n.s., not significant, using one-way ANOVA with Tukey’s *Post hoc* test for multiple comparisons.

We also assessed the gross morphology of abdominal NMJs with muscle-specific knockdown of Dystrophin (Dietzl et al., 2007). Although we did not identify significant changes in bouton number between *dys*^*Det*–1^ mutants and WT controls, we did identify a significant reduction in bouton number using muscle-specific knockdown of Dystrophin (Figures 7A–E). Knockdown of Dystrophin resulted in decreased bouton number even at Day 5, which continued at Day 21. While bouton number changed upon RNAi-mediated knockdown of Dystrophin, bouton size was not impacted. There were no significant differences in bouton size with knockdown of Dystrophin in muscles compared to controls (Figure 7F). These results are consistent with those found using *dys*^*Det*–1^ mutants (Figure 3).

**FIGURE 8:**
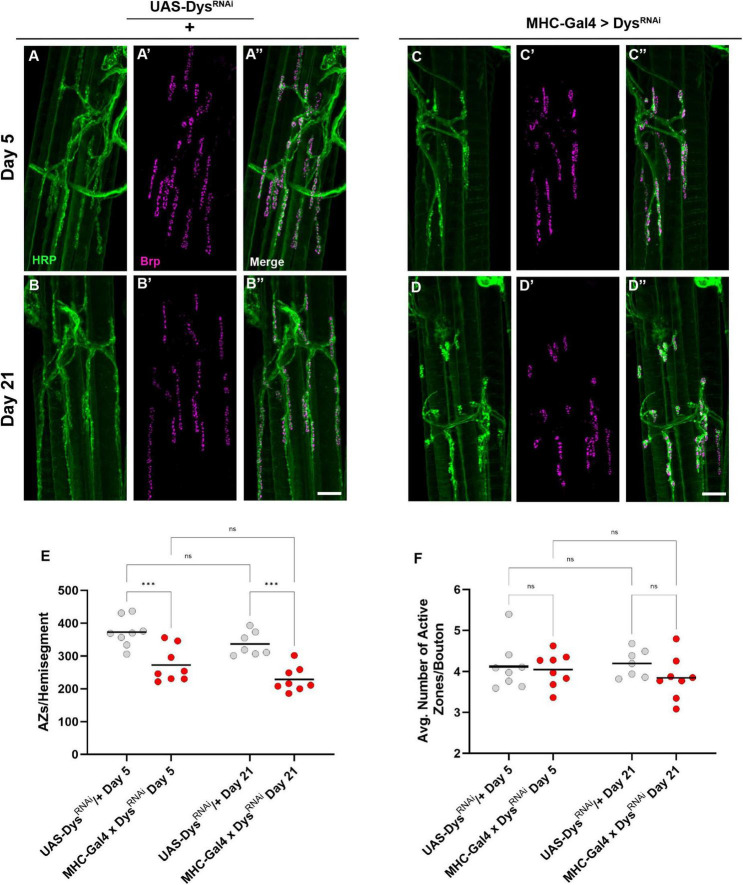
Dystrophin expression is required in muscles to maintain active zone distribution. **(A–D)** Active zone distribution at abdominal NMJs in *UAS/* + controls **(A,B)** and flies with muscle-specific knockdown of Dystrophin **(C,D)**. Active zones are designated by Brp + puncta (magenta). **(E)** Average number of active zones located within each individual muscle (hemi-segment) muscle-specific knockdown of Dystrophin (red dots) compared to *UAS/* + controls (gray dots) at Day 5 and Day 21. **(F)** Average number of active zones within each synaptic bouton at Day 5 and Day 21. Scale Bar in panel **(D)** is 10 μm for panels **(A–D)**. ***p < 0.001; n.s., not significant, using one-way ANOVA with Tukey’s *Post hoc* test for multiple comparisons.

We next measured Brp staining upon muscle-specific knockdown of Dystrophin to assess active zone distribution (Figure 8). At Day 5 fewer boutons per NMJ were detected following knockdown of Dystrophin. Similar to *dys*^*Det*1^ mutants (Figure 4), this phenotype persisted at Day 21 (Figures 8A–E). However, the average number of active zones within each bouton remain unchanged with knockdown of Dystrophin (Figure 8F). This is likely explained by the decrease in bouton number resulting from muscle-specific knockdown of Dystrophin (Figure 7). These results suggest that the loss of active zones parallels the loss of boutons, while the remaining boutons contain the appropriate number of active zones.

**FIGURE 9:**
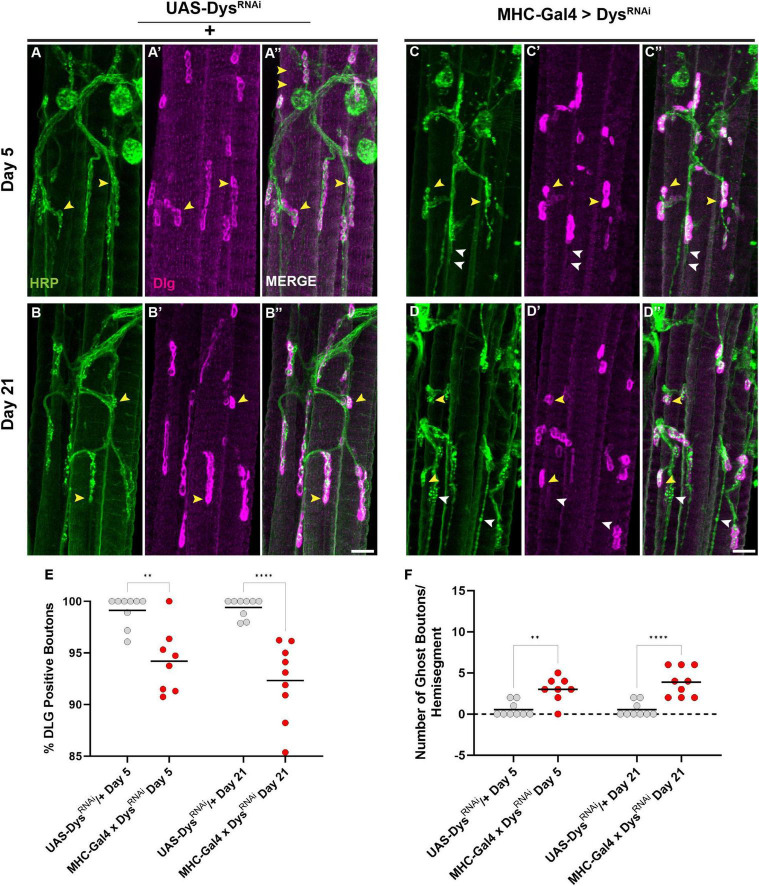
Dystrophin expression is required in muscles to maintain the subsynaptic reticulum. **(A–D)** DLG labels the subsynaptic reticulum (magenta) at abdominal NMJs in *UAS/* + controls **(A,B)** and flies with muscle-specific knockdown of Dystrophin **(C,D)**. **(E)** Percentage of HRP + boutons that are also DLG + (yellow arrowheads) in *UAS/* + controls (gray dots) compared to muscle-specific knockdown of Dystrophin (red dots) at Day 5 and Day 21. **(F)** Prevalence of ghost boutons, which are defined as HRP + but DLG- boutons (white arrowheads) in *UAS/* + controls (gray dots) compared to muscle-specific knockdown of Dystrophin (red dots) at Day 5 and Day 21. Scale Bar in panel **(D)** is 20 μm for panels **(A–D)**. ****p < 0.0001; **p < 0.01; n.s., not significant, using one-way ANOVA with Tukey’s *Post hoc* test for multiple comparisons.

Finally, we assessed how muscle-specific knockdown of Dystrophin impacted postsynaptic structures such as the SSR. We measured the percentage of synaptic boutons that were surrounded by an intact SSR using anti-DLG staining. Upon muscle-specific knockdown of Dystrophin, we found a decrease in boutons surrounded by DLG staining even at Day 5 (Figures 9A–E). This decrease in DLG + boutons became even greater by Day 21 (Figures 9B–E). As with our mutant analysis, we also measured the number of ghost boutons upon muscle-specific knockdown of Dystrophin. At Day 5, knockdown of Dystrophin resulted in a significant increase in the average number of ghost boutons compared to controls, and this value increased further by Day 21 (Figure 9F). Together, these results demonstrate that muscle-specific knockdown of Dystrophin impairs both pre-and post-synaptic integrity at adult NMJs.

## 3 Discussion

Our results highlight the synaptic impairments seen in adult NMJs using a model of Muscular Dystrophy. The progressive loss of flight ability in *dys*^*Det*–1^ mutants is accompanied by impaired structural integrity of NMJs in the indirect flight muscles, indicating the role of Dystrophin in maintaining both functional and structural integrity of synapses in aging flies. We also identified deficits in both presynaptic and postsynaptic structures within flight muscles as well as abdominal muscle NMJs. Although recent studies have identified broader roles for Dystrophin beyond maintaining muscle fiber integrity, our results using tissue-specific knockdown of Dystrophin indicate that Dystrophin is specifically required in the muscle to maintain synaptic integrity. Interestingly, post-synaptic expression of Dystrophin is even required to maintain presynaptic integrity.

While the muscle-specific knockdown of Dystrophin largely recapitulated the phenotypes observed in *dys*^*Det*–1^ mutants, we did identify some phenotypic differences. For example, knockdown of Dystrophin in muscles resulted in a decrease in bouton number at abdominal NMJs (Figure 7). However, we did not find a similar decrease in bouton number in *dys*^*Det*–1^ mutants (Figure 3). There are several possible explanations for this difference in phenotype. One possible reason for the more robust phenotype using RNAi is that the level of knockdown with these transgenes is greater than the loss of function using the hypomorphic *dys*^*Det*–1^ allele (Christoforou et al., 2008). The phenotypic variance could also be due to differences in genetic background between mutant and RNAi stocks, since genetic background can have an impact on synaptic maintenance (Sidisky et al., 2023).

One of the most striking findings from the current study is the that the knockdown of Dystrophin in postsynaptic muscle cells impairs presynaptic as well as postsynaptic structure. Muscle-specific knockdown of Dystrophin not only impairs presynaptic morphology (Figure 7), but also active zone distribution in motor neuron terminals (Figure 8). This may suggest that trans-synaptic signaling is specifically impaired in Muscular Dystrophy. Indeed, trans-synaptic Bone Morphogenetic Protein (BMP) signaling is well documented as a regulator of synaptic development, growth, and maintenance (Marques et al., 2002; McCabe et al., 2003; Sidisky et al., 2021). It is also important to consider the specific defects to muscle architecture and how they could relate to the synaptic defects seen in the current study. *dys*^*Det*–1^ mutants have decreased levels of the Titin homolog Projectin within sacromeres at Day 3-5, which becomes more prominent by Day 13-15 (Pantoja et al., 2013). Since these changes are noticed in early adults, they could occur in parallel with, or even precede, many of the synaptic impairments described here.

We also cannot rule out a possible role for Dystrophin within motor neurons or glial cells in maintaining synaptic integrity. While we did not detect any changes to NMJ structure or function upon knocking down Dystrophin in these tissues individually, it is certainly possible that knockdown in multiple tissues is required to uncover a phenotype. Future studies using this method, along with tissue-specific rescue of Dystrophin in a mutant background, should help to further highlight the role(s) of Dystrophin at the tripartite synapse.

It will also be interesting to compare the synaptic defects shown here in a model of Muscular Dystrophy with those of neurodegenerative diseases. Synaptic defects have been demonstrated in every major neurodegenerative disease, and these synaptic impairments currently represent the earliest known hallmarks of these diseases (Selkoe, 2002; Milnerwood and Raymond, 2010; Picconi et al., 2012; Shahidullah et al., 2013). Are the mechanisms that underlie the earliest stages of pathology shared between Muscular Dystrophy and neurodegenerative diseases? If so, then understanding synaptic defects in these diseases could have broad implications for identifying potential therapeutic targets for diseases that currently lack a cure.

## 4 Materials and methods

### 4.1 Fly stocks and husbandry

Fly stocks were raised and maintained on standard Drosophila media at 25°C. Upon eclosion, flies used for experimental analysis were collected and raised to the designated age at 29°C.

The following fly stocks used in this study were received from the Bloomington Drosophila Stock Center: *dys*^*Det*1^ (#63046) (Christoforou et al., 2008), *Oregon-R* (#5), *MHC-Gal4* (#55132) (Klein et al., 2014), *OK371-Gal4* (#26160) (Meyer and Aberle, 2006), *Repo-Gal4* (#7415) (Sepp et al., 2001), *UAS-Dys^RNAi^* (#31553) (Ni et al., 2011). The following stock was obtained from the Vienna Drosophila Resource Center: *UAS-Dys^RNAi^* (#106578) (Dietzl et al., 2007).

### 4.2 Flight behavior

Flight ability was measured by dropping flies into a 90-cm polypropylene cylinder as previously described (Babcock and Ganetzky, 2014). Flies were dropped from vials into the large cylinder with the walls coated in Tangle Trap (Tanglefoot Company). The landing height within the cylinder was recorded to the nearest centimeter for each individual fly. For analysis, the landing height of each fly as well as the group average was measured for each condition.

### 4.3 Immunohistochemistry

Dorsal Longitudinal Muscles were dissected as previously described (Sidisky and Babcock, 2020). Briefly, fly legs are removed from the ventral side of the thorax using dissection scissors. Thorax samples are then fixed for 30 min in 4% formaldehyde. Fixed samples are flash frozen in liquid nitrogen, and each thorax is bisected using a razor blade. Bisected samples are then transferred to 2.0 mL microtubes for antibody staining.

Abdominal dissections were prepared by removing the abdomen from the thorax. A cut was made at the distal tip of the abdominal segment and along the dorsal midline of the abdomen (Hebbar et al., 2006). Insect dissection pins were then used to secure the abdominal filet and the internal viscera was removed to expose the ventral abdominal NMJs. Preps were fixed in the dissection dish for 30 min in 4% PFA in 1XPBS. Preps were then washed 4x with PBS that includes 0.3% Triton X-100 (0.3% PBST) prior to antibody treatments.

The DLM tissue samples were incubated in blocking buffer made with 0.2% PBST and 1.0% BSA for 24 h before primary antibody treatment and incubated in primary antibodies for 48 h at 4°C. Abdominal muscle preps were treated with 0.1% Normal Goat Serum Blocking Buffer in 0.2% PBST for at least 1hr then treated with primary antibodies for 24 h at 4°C. Primary antibodies from the Developmental Studies Hybridoma Bank include mouse anti-Bruchpilot (nc82) (1:25) and mouse anti-Disks Large (4F3) (1:500). Secondary antibodies include goat anti-mouse Alexa-568 (1:200) (Invitrogen). Samples were incubated in secondary antibody (as well as FITC-HRP) for 2 h at room temperature in the dark. Neuronal membranes were stained using FITC-conjugated Horseradish Peroxidase (HRP) (1:200) (Jackson Laboratories). Samples were then washed with 0.3% PBST 4x and mounted on glass slides with Vectashield (Vector Laboratories). DLM tissues were mounted as previously described (Sidisky et al., 2021), and abdominal tissue was mounted with 1 layer of reinforcements prior to addition of the glass cover slip.

### 4.4 Image acquisition

All images were acquired using a Zeiss LSM 880 confocal microscope equipped with a 63x objective. For DLM tissue samples, Z-stacks were created using 45 slices with a 0.7 μm interval for gross morphology. For consistency, all images were acquired along the posterior portion of muscle fiber C beginning at the muscle surface. Images for DLM presynaptic markers were acquired with a 63x objective with a 2.5x zoom and transformed into a Z -stack of 30 slices with a 1.0 μm interval of muscle fiber C. For analysis, 15 images were acquired, 3 per sample, on the anterior region of muscle fiber C, over 5 independent samples for each condition for an accurate assessment of the synaptic markers. For VAM tissue samples, Z-stacks were created using 40 slices with a 1.0 μm interval with a 1.2x zoom. All images were taken from abdominal segment A3 for consistency. Maximum intensity projections were created from each Z-stack using FIJI. Brightness and contrast were adjusted equally for each sample group using Adobe Photoshop CC2023 and ImageJ software FIJI (Schindelin et al., 2012).

### 4.5 Image analysis

For assessment of gross morphology of DLM NMJ preps a samples size of 10 images were analyzed for each condition. Total neurite length (μM) from Z-stacks were measured using the Simple Neurite Tracer (SNT) plug-in to trace the HRP staining (Longair et al., 2011; Arshadi et al., 2021) and analyzed using the Skeletonize 3D plug-in (Schindelin et al., 2012) as previously described (Sidisky et al., 2021, 2023). Boutons were counted manually for each image using the Cell Counter tool in Fiji (Schindelin et al., 2012). The ratio of bouton number to total neurite length was attained by dividing the bouton number by total neurite length for each image.

The presynaptic active zones were assessed by using BRP as the active zone marker and counting the number of boutons with a sample size of 15 images. Boutons that had BRP staining within the bouton area were counted as BRP positive. This was calculated by dividing the number of BRP positive boutons by the total number of boutons and multiplied by 100 to get a percentage for each image and condition. The number of active zones per bouton was assessed by manually counting the number of BRP puncta at each bouton and taking the average of each active zone per bouton for each image and condition.

To assess the gross morphology of the VAM NMJs, for each condition, at least six hemi-segments across 5 samples were analyzed. Bouton number was counted manually using the Cell Counter tool and bouton area for each bouton was traced measured by using the oval tool in Fiji to access the average bouton area across each sample and condition.

The presynaptic active zones of VAM NMJs were assessed by counting the total number of BRP puncta per hemi-segment using the Cell Counter Fiji plug-in using the HRP to identify the presynaptic bouton. The average number of active zones per bouton was counted for each bouton traced with the oval tool and the Region of Interest (ROI) tool and taken as an average for each image and condition. To assess the postsynaptic muscle tissue, DLG was used along with HRP to identify the presynaptic bouton. First, for each image the outer perimeter of each bouton was traced using the oval tool along with the ROI tool Going slice by slice through the Z-stack, for each bouton, the presence of absence of DLG was recorded using the HRP stain to detect the presynaptic bouton. The percentage of DLG positive boutons was calculated by taking the number of DLG positive boutons by the total number of boutons per hemi-segment and multiplied by 100 to get a percentage for each image and condition. The number of ghost boutons was assessed as a presynaptic bouton without any postsynaptic DLG staining (Guangming et al., 2020) for each image and condition.

### 4.6 Statistical analysis

All measurements of synaptic morphology were analyzed using a one-way ANOVA along with Tukey’s *post hoc* analysis for multiple comparisons. GraphPad Prism 9 (GraphPad Software) was used to carry out all statistical analysis and generate graphs. All quantified data is displayed with all individual data points along with the mean value for each group.

## Data availability statement

The original contributions presented in this study are included in the article/supplementary material, further inquiries can be directed to the corresponding author.

## Ethics statement

The manuscript presents research on animals that do not require ethical approval for their study.

## Author contributions

JS: Conceptualization, Formal analysis, Investigation, Methodology, Writing – original draft, Writing – review and editing. AW: Formal analysis, Investigation, Methodology, Writing – original draft. RC: Formal analysis, Investigation, Methodology, Writing – original draft. DB: Conceptualization, Formal analysis, Funding acquisition, Investigation, Project administration, Supervision, Writing – original draft, Writing – review and editing.
